# Editorial on Special Issue: “Novel Therapeutic Strategies for Glioblastoma”

**DOI:** 10.3390/pharmaceutics17070815

**Published:** 2025-06-24

**Authors:** Ana Podolski-Renić, Jelena Dinić, Milica Pešić

**Affiliations:** Institute for Biological Research “Siniša Stanković”—National Institute of the Republic of Serbia, University of Belgrade, Despota Stefana 142, 11108 Belgrade, Serbia; ana.podolski@ibiss.bg.ac.rs (A.P.-R.); jelena.dinic@ibiss.bg.ac.rs (J.D.)

## 1. Introduction

Glioblastoma represents the most prevalent and most aggressive form of primary malignant brain tumor in adults. It is composed of different cell populations with varying genetic and phenotypic profiles, which contributes to its aggressive behavior and poor prognosis. A hallmark of glioblastoma is its infiltrative nature and invasion to surrounding brain tissue. In addition, glioblastoma cells exhibit intrinsic resistance to a variety of therapeutic interventions, including conventional chemotherapy, radiation, and new treatment modalities. Over the past two decades, a variety of clinical trials and therapeutic strategies—from targeted molecular therapies and cell-based approaches to immunotherapies —have been tested, but none has shown a significant improvement in overall survival. The current standard treatment involves maximum safe surgical resection of the tumor mass, followed by radiotherapy in combination with temozolomide therapy. Although this multimodal approach can temporarily delay tumor progression, it does not ultimately lead to a significant extension of median survival beyond 15–18 months. Therefore, there is an urgent need for novel therapeutic strategies that are able to overcome the resistance mechanisms and adaptive capabilities of glioblastoma. Such innovations are critical to improve the survival rates of glioblastoma patients. 

## 2. Overview of the Published Articles

This Special Issue assembles six original articles dealing with the molecular basis and therapeutic vulnerabilities of glioblastoma. Topics include the molecular links between glioblastoma, ischemic stroke, and moyamoya disease, as well as advances in the nanoparticle delivery of temozolomide and the identification of new biomarkers. Other studies focus on the modulation of immunosuppression, targeting glioma stem cell metabolism, and overcoming multidrug resistance (MDR) through combination therapies.

New findings indicate that there are significant pathological and molecular links between glioblastoma, ischemic stroke, and moyamoya disease, but these have not yet been sufficiently explored. In a study by Islam et al., a comprehensive bioinformatics approach was used to identify common gene expression patterns, signaling pathways, and protein–protein interactions in the three diseases. The analysis uncovered new transcription factors and microRNAs that suggest potential molecular links and offer novel insights into the shared mechanisms of disease development and progression.

Solid lipid nanoparticles (SLNPs) have shown promise as carriers for various chemotherapeutics, but their use with temozolomide (TMZ) in resistant glioblastoma remains poorly understood. Nasir et al. evaluated SLNP-conjugated TMZ (SLNP-TMZ) for targeted drug delivery in TMZ-sensitive and -resistant glioblastoma models. Compared to free TMZ, SLNP-TMZ significantly enhanced cytotoxicity and decreased cell migration in vitro and effectively reduced tumor burden in an orthotopic xenograft mouse model. Notably, SLNP-TMZ enabled higher accumulation of the drug in brain tissue while minimizing systemic exposure, thereby reducing off-target toxicity. Furthermore, the increased expression of inflammatory markers (IL-1β, IL-6, and TNF-α) in resistant models underscores the therapeutic challenges of TMZ resistance—challenges that can be overcome by SLNP-based delivery.

A study by Pokorná et al. investigated the potential of prominin-1, intercellular adhesion molecule-1 (ICAM-1), and the long non-coding RNAs PARTICLE and GAS5 as diagnostic and prognostic biomarkers in glioblastoma. Using histological and molecular techniques—including hemoxylin and eosin staining, immunofluorescence, in situ hybridization, and qPCR—this study identified different expression patterns that correlate with tumor grade and response to therapy. Prominin-1 was localized in pseudo-palisade regions, ICAM-1 was associated with vascular degeneration, and nuclear PARTICLE expression indicated tumor-promoting activity. GAS5, which was enriched in necrotic regions, was associated with improved survival, supporting its role as a tumor suppressor. These results emphasize the potential of the analyzed biomolecules to improve glioblastoma detection, treatment stratification, and prognosis.

Glioma stem cells (GSCs) are a subpopulation of self-renewing, therapy-resistant cells that drive tumor growth. Although the resistance of GSCs to conventional therapies is well characterized, their resistance to tyrosine kinase inhibitors (TKIs) remains poorly understood. Aldaz et al. explored adaptive mechanisms of GSCs in response to ponatinib, a multi-targeting TKI, using compartmentalized proteomics. The study showed that GSCs undergo a robust metabolic adaptation characterized by rewiring of lipid metabolism—specifically increased fatty acid β-oxidation, cholesterol biosynthesis and sphingolipid degradation. The pharmacologic inhibition of these metabolic pathways effectively reversed ponatinib resistance in vitro. Most importantly, an analysis of patient-derived data underscored sphingolipid degradation as a clinically relevant hallmark in GBM. These results position lipid metabolism—and sphingolipid degradation in particular—as a promising therapeutic vulnerability in GSCs.

The tumor microenvironment of IDH-wildtype glioblastoma is dominated by myeloid-derived suppressor cells (MDSCs), but the mechanisms driving their suppressive phenotype remain unclear. Recent findings show that MDSCs in glioblastoma express various Sialic acid-binding immunoglobulin-like lectin (Siglec) receptors that can transmit inhibitory signals comparable to PD-1 and act as glyco-immune checkpoints. Cornelissen et al. showed that glioblastoma cells induce an immunosuppressive profile in monocytes via Siglec–sialic acid interactions. The desialylation of glioblastoma cells impaired the upregulation of CD163 and enhanced the secretion of the proinflammatory cytokines IL-6 and TNFα by monocytes, suggesting a reversal of the suppressive state. In addition, the direct activation of Siglec-7 and Siglec-9 decreased the production of pro-inflammatory cytokines, further confirming the role of the Siglec–sialic acid axis in immune regulation. These findings suggest that targeting tumor-associated sialylation may represent a novel strategy to counteract myeloid immunosuppression and improve therapeutic outcomes in glioblastoma.

A study by Podolski-Renić et al. investigated the potential of LB-100, a small molecule inhibitor of protein phosphatase 2A, in combination with adavosertib (a WEE1 kinase inhibitor) and doxorubicin (DOX) to overcome MDR in cancer cells and improve treatment efficacy. LB-100 enhanced the effectiveness of adavosertib and DOX after multiple applications in patient-derived glioblastoma and non-small cell lung carcinoma (NSCLC) cells. LB-100 reduced P-glycoprotein levels and altered the transcriptional dynamics of genes associated with treatment response in MDR glioblastoma and NSCLC cells. In addition, LB-100 increased the accumulation of DOX and its cytotoxicity in MDR cancer cells, especially when administered in a subsequent treatment. 

## 3. Conclusions and Future Directions

In conclusion, this Special Issue highlights significant advancements in understanding the molecular mechanisms underlying glioblastoma and therapeutic vulnerabilities that can be exploited for more effective treatments. The research presented emphasizes the importance of exploring novel biomarkers, enhancing drug delivery systems like solid lipid nanoparticles, and addressing treatment resistance in glioblastoma through innovative strategies.

Future directions should prioritize validating biomarkers for clinical use and exploring combination therapies. Investigating immune suppression in glioblastoma could enhance immunotherapy effectiveness. The continued development of targeted nanoparticles and small-molecule inhibitors may transform treatment paradigms and improve patient outcomes in glioblastoma and other resistant cancers. Collaborative research that combines bioinformatics, molecular biology, and clinical applications will be essential for creating more personalized and effective glioblastoma treatments.

